# Monitoring the Spoilage of Fresh Sterlet (*Acipenser ruthenus*) During Storage at 4 °C by Mid-Infrared and Fluorescence Spectroscopies Coupled with Chemometric Tools

**DOI:** 10.3390/foods14122051

**Published:** 2025-06-11

**Authors:** Daria Vilkova, Moriken Sangaré, Ahmed Snoussi, Romdhane Karoui

**Affiliations:** 1 Univ. Artois, Univ. Lille, Univ. Littoral Côte d’Opale, Univ. Picardie Jules Verne, Univ. de Liège, INRAE, Junia, UMR-T 1158, BioEcoAgro, F-62300 Lens, France; dariavilkova333@gmail.com (D.V.); morikens@yahoo.fr (M.S.); 2 Department of Biotechnology, Aquaculture, Soil Science and Land Management, Univ. Astrakhan State, Astrakhan R-414056, Russia; 3 Laboratory of Innovation and Valorization for a Sustainable Food Industry, Higher School of Food, Industries of Tunis, University of Carthage, LR21AGR04, 58 Avenue Alain Savary, Tunis 1003, Tunisia; ahmed.snoussi@esiat.ucar.tn

**Keywords:** Sterlet (*Acipenser ruthenus*), freshness, fluorescence, infrared, spoilage

## Abstract

Sterlet is a perishable product; therefore, its freshness monitoring and shelf-life evaluation are important. In this study, a series of analytical techniques named physicochemical, microbiological, sensory, colorimetric, and mid-infrared and fluorescence spectroscopies were applied on Sterlet (*Acipenser ruthenus*) samples during 18 days of storage at 4 °C. The water content increased from 72.8 g/100 g on day 1 to 77.81 g/100 on day 14. Regarding the peroxide value (PV), the initial value was 4.17 meq/kg of Sterlet on day 1, reaching a maximum on day 4 (4.9 meq/kg of Sterlet), and then it decreased gradually, attaining a value of 0.7 meq/kg of Sterlet on day 18. Generally, the thiobarbituric acid reactive substance (TBARS), total viable count (TVC) and psychrotrophic count (PTC) increased during the storage time and increased from 0.03 to 0.13 MDA eq./kg of Sterlet sample, 2.27 to 9.09 log10 CFU/g, and 2.18 to 9.15 log10 CFU/g, respectively, on day 1 and 18, respectively. The microbiological and sensory analyses indicated that Sterlet samples were acceptable for human consumption up to 7 days of storage at 4 °C. This result was confirmed by fluorescence measurements, since the principal component analysis (PCA) applied to the NADH and MIR spectra allowed for a clear differentiation between Sterlet samples aged 7 days or less from the others. This trend was confirmed by the factorial discriminant analysis (FDA) applied to the NADH and MIR spectra, since a correct classification with leave-one cross-validation of 94.44% was observed. In addition, the heatmap of the Pearson correlation coefficients showed high correlations between overall acceptability and microbiology parameters and the structural properties of Sterlet samples during storage, indicating that the modifications observed at the macroscopic level were related to those notedat the molecular scale.

## 1. Introduction

Acipenseriformes includes two extant families, with Acipenseridae comprising 25 of the species. Among them, we can enumerate Beluga Sturgeon (*Huso huso*), White Sturgeon (*Acipenser transmontanus*), Sterlet Sturgeon (*Acipenser ruthenus*), Sevruga (*Acipenser stellatus*), Russian Sturgeon (*Acipenser gueldenstaedtii*), and Polyodontidae, which are composed of two species, namely Mississippi paddlefish (*Polyodon spathula*) and Chinese paddlefish (*Psephurus gladius*) [1]. Sturgeons are located from the subtropical to the subarctic rivers and lakes of Eurasia and North America. Habitat destruction, lack of river connectivity, pollution, and illegal overfishing have led most sturgeon species into a threatened status [2]. The aquaculture of sturgeon species has expanded worldwide due to the international demand for their flesh and caviar for human diets [3]. By 2017, the production of sturgeon biomass reached around 103 tons and there were 2329 commercial sturgeon farms [4]. The Sterlet (*Acipenser ruthenus*) represents the smallest sturgeon species that inhabits freshwater. Among other sturgeon species, Sterlet is also rich in polyunsaturated fatty acids including omega-3/omega-6 essential fatty acids [5].

Freshness is the most relevant attribute that consumers use for selecting fish and fish products. However, freshness is subject to fast changes during storage due to the biochemical reactions, the presence of autolytic enzymes, and the microbiological activity that changes their appearance, odour, colour, taste, and texture [6]. In this context, sensory analysis is used as a valuable technique for the determination of the freshness state and shelf-life of fish. The quality index method, related to the determination of quality parameters developed for each fish species, was used to predict the organoleptic properties and fish quality [7].

To monitor fish freshness, other techniques, such as electronic tongue with metallic potentiometric electrodes [8], enzyme electrode sensors with an injection system [9], and a portable electronic nose system [10], have been used. Microbiological analyses based on the determination of total viable counts (TVCs), psychrotrophic counts (PTCs) [11], and chemical analyses centred on the determination of the peroxide value (PV) and thiobarbituric acid reactive substance (TBARS) values [12] have been applied to determine the freshness level of fish. However, most of the aforementioned techniques are destructive, expensive, time-consuming, and need skilled operators. Spectroscopic techniques including nuclear magnetic resonance (NMR), fluorescence, mid-infrared, and near-infrared are increasingly used for the determination of the quality of fish and fish products [13,14,15]. Front-face fluorescence spectroscopy (FFFS) has been applied to monitor the freshness state of different fish species, such as Russian sturgeon (*Acipenser gueldenstaedtii*) [16], Whiting fish (*Merlangius merlangus*) [17], Atlantic mackerel (*Scomber scombrus*), Sea bass (*Dicentrarchus labrax*) [18,19], and so on.

However, most of the above-mentioned research studies which explored rapid analytical techniques coupled with chemometric tools to determine the quality of different fish species during storage were performed without using sensory and microbiological analyses. Thus, this study aimed, to the best of our knowledge for the first time, to investigate the potentiality of different analytical techniques, namely physicochemical, colour, sensory, microbiological, and fluorescence and mid-infrared spectroscopies, to monitor the freshness state and to determine the shelf-life of Sterlet during storage at 4 °C. The techniques proposed in this study offer the implementation of a multidimensional approach for monitoring the quality of Sterlet (*Acipenser ruthenus*) during storage at 4 °C. This multidisciplinary approach maximises the reliability of the results by crossing direct (microbial) and indirect (spectral) measurements following the determination of correlation by using the heatmap of the Pearson correlation, and therefore reducing the individual limitations of each method (sensory subjectivity, slowness of microbiological analyses).

## 2. Materials and Methods

### 2.1. Chemicals and Reagents

To conduct this research study, the chemical products are of analytical grade. The 2-thiobarbituric acid (99%), trichloroacetic (99.5%), sodium thiosulphate, potassium iodide, and acetic acid are obtained from MERCK (Darmstadt, Germany). Hexane, chloroform, isopropanol, sodium sulphate anhydrous, and Celite^®^ 545 are purchased from VWR International (Rosny Sous Bois, France).

### 2.2. Fish Sample Preparation 

Four-year-old Sterlet (*A. ruthenus*) were collected from the fish farm located in Astrakhan (Russia). The Sterlet samples were fed a group of vitamins, wheat flour, flour, sunflower oil, fish oil, and mono- and calcium phosphate. The Sterlet samples were killed by asphyxiation/hypothermia in the Aquatrade farm and stored on ice during transport. Upon arriving at the laboratory, the samples were first beheaded, eviscerated, and processed to obtain Sterlet slices, and then packed in partially vacuum-sealed plastic bags. A total of twenty-four (24) samples were kept at 4 °C and randomly analysed on day 1, 4, 7, 11, 14, and 18.

### 2.3. Microbiological Analysis

Total viable counts (TVCs) and psychrotrophic counts (PTCs) were determined based on the method described by Ojagh, Rezaei, Razavi, & Hosseini [20] with some modifications. A total of 10 g of the Sterlet samples was homogenised in 90 mL NaCl solution prepared at 0.85%. Decimal dilutions were realised and inoculated in plate count agar media (PCA, Merck, Darmstadt, Germany) by using the pour plate method. The inoculated plates were incubated at 37 °C over 2 days for TVC, and at 10 °C over 7 days for PTC. All counts were expressed as log10 CFU g^−1^ and performed in triplicate.

### 2.4. Sensory Analysis

In the sensory evaluation of fish raw material, the most sensitive indicators are odour, colour and texture. Changes in these characteristics are associated with a biochemical alteration. The appearance and surface colour of the samples were assessed through visual inspection. The sensory evaluations of Sterlet samples during storage were performed as described by Zhang et al. [21]. The panel consisted of eight trained members (aged 27–45 years) and analyses were performed blindly in a laboratory equipped with individual evaluation booths under controlled light conditions (white fluorescent lamps). Samples were coded so that the evaluation was performed blindly. Each evaluator was asked to evaluate colour, texture, odour, and overall acceptability. The colour of the Sterlet samples was evaluated on the surface at a cross-section made immediately during examination, while the texture was determined by pressing a finger onto the fish’s surface or a cross-section. The fish’s odour was assessed on the surface. A 1–10-point rating scale was used, with 10 being the highest quality and 1 indicating the limit of acceptability: colour (10, no discoloration; 1, extreme discoloration); texture (10, firm; 1, very soft); odour (10, extremely desirable; 1, extremely unacceptable/unpleasant odour) and overall acceptability (10, extremely desirable; 1, extremely unacceptable) of the samples. The scores for the different characteristics were added together to obtain an overall sensory score. Thus, the maximum total score was 40 points (perfectly fresh fish samples), and depending on the degree of spoilage, the score decreased. A total score of less than 24 based on the indicators of odour, colour, texture, and overall acceptability was considered the lower acceptable limit of suitability. This threshold was based on the fact that score values less than 24 were ascribed for altered samples based on our preliminary results.

### 2.5. Measurement of Physicochemical Properties

Water content was determined according to the AOAC [22]. The peroxide value (PV) was determined by iodometric titration by applying the method employed by Eymard [23]. The thiobarbituric acid reactive substance (TBARS) value was performed according to the method employed by Guizani, Rahman, Al-Ruzeiqi, Al-Sabahi, & Sureshchandran [12].

### 2.6. Colour Measurements

The colour of the Sterlet samples was assessed by using the Minolta Chroma Meter version CR-300 (Konica Minolta Sensing Europe, Roissy-en-France, France). Measurements of L*, a*, and b* were performed directly on Sterlet samples. The total colour difference (ΔE*) between 1-day-old Sterlet samples (considered as the reference) and the other samples was calculated as follows:

$${\Delta E}^{*}=\sqrt{{\left(L^{*}-L_{0}\right)}^{2}+{\left(a^{*}-a_{0}\right)}^{2}+{\left(b^{*}-b_{0}\right)}^{2}}$$

The whiteness (*WI*) and yellowness (*YI*) indices were determined as follows:

$$WI=100-\sqrt{{\left(100-L^{*}\right)}^{2}+{\left(a^{*}\right)}^{2}+{\left(b^{*}\right)}^{2}}$$

$$YI=142.86\;(\frac{b*}{L*})$$

### 2.7. Mid-Infrared Spectroscopy Measurements

The mid-infrared (MIR) spectra of Sterlet samples were acquired between 4000 and 700 cm^−1^ with the Fourier transform spectrometer IRTracer-100 (Shimadzu, Noisiel, France). An attenuated total reflection (ATR) accessory equipped with a grip (Pike Technologies, Inc., Madison, WI, USA) was used. The ATR cell presented the following characteristics: It was a ZnSe crystal with a total reflection of 10 and an incidence angle of 45°. To assure good contact between the ZnSe crystal and Sterlet sample, gentle pressure was applied. Before each measurement, a background was acquired by using the ZnSe crystal. The ZnSe crystal was carefully cleaned using ethanol and ultra-pure water for each measurement.

To determine the secondary structure of Sterlet samples during storage at 4 °C, the Amide I band (1700–1600 cm^−1^) was used by applying a derivative procedure that provided more accurate information about changes in the secondary structure of proteins during Sterlet storage. The derivative procedure was applied using LabSolutions Software (LabSolution, IR 2.31).

### 2.8. Fluorescence Spectroscopy Measurements

Fluorescence spectra were scanned with a Fluoromax-4 spectrofluorimeter (Jobin Yvon, Horiba, NJ, USA). The spectrofluorimeter was equipped with a thermostatic cell and the temperature controlled by a Haake A25, AC 200 temperature controller (Thermo-Scientific, Courtaboeuf, France). Sterlet samples approximately 2 cm in length, 1 cm in width, and 0.5 cm in thickness were cut and mounted between two quartz slides. The emission spectra of tryptophan (305–450 nm), nicotinamide adenine dinucleotide (NADH) (360–600 nm), and riboflavin (405–650 nm) were scanned after excitation set at 290, 340, and 380 nm, respectively. The vitamin A excitation spectra (250–390 nm) were acquired after emission set at 410 nm.

### 2.9. Statistical Analysis

To monitor the Sterlet freshness state throughout storage, the Bonferroni test was applied as part of the one-way analysis of variance (ANOVA) of the chemical, textural, and colour parameters. The correlation between some specific parameters and storage time was determined.

The principal component analysis (PCA) was performed on the normalised physicochemical, colorimetric, microbiological, mid-infrared, and fluorescence data sets [24,25]. The normalised data sets were analysed separately by principal component analysis (PCA), an unsupervised tool aimed at representing a large number of variables and individuals in the form of graph [24]. In the second step, factorial discriminant analysis (FDA) was applied on the five PCs resulting from the PCA applied to the MIR and fluorescence spectra [24]. The first five PCs of the PCA, applied to each fluorophore and MIR spectra representing more than 99% of the total variance, were analysed by factorial discriminant analysis (FDA) with leave-one-out cross-validation [25]. Six groups were defined before applying the FDA: samples aged 1, 4, 7, 11, 14, and 18 day(s). A comparison of the number of samples belonging to a specific group vs. the real one gave information about the robustness of the discrimination. Taking into account the low number of samples analysed for each storage time, FDA with cross-validation was applied, allowing the sample to be used both for calibration and prediction models.

ANOVA and FDA were performed with XLSTAT 2014 (Addinsoft SARL USA, New York, NY, USA) software, while PCA was determined by using MATLAB software (2014a) (The MathWorks, Natick, MA, USA).

## 3. Results and Discussion

### 3.1. Microbiological Analysis

**Figure 1:**
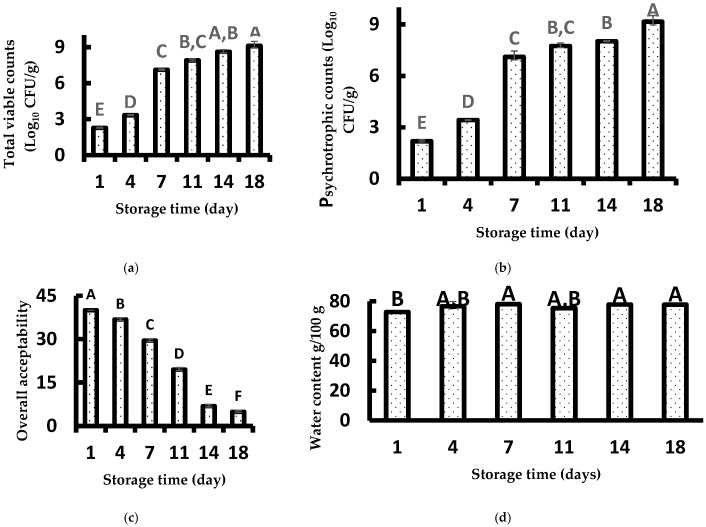

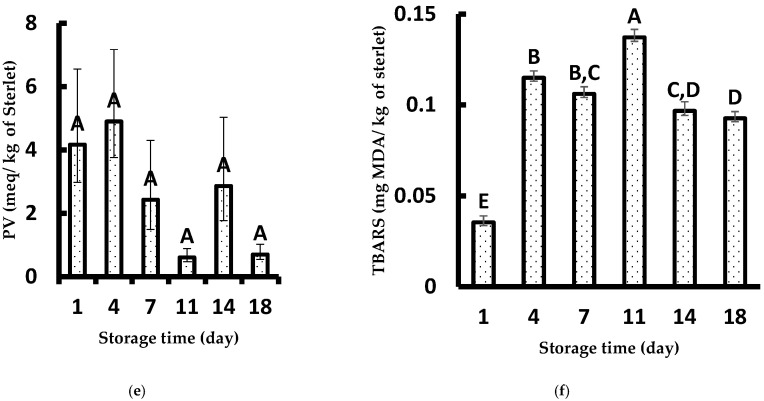
Evolution of (**a**) total viable counts (TVCs), (**b**) psychrotrophic counts (PTCs), (**c**) sensory analysis, (**d**) water content, (**e**) peroxide value (PV) and (**f**) thiobarbituric acid reactive substances (TBARS) performed on Sterlet samples stored at 4 °C for up to 18 days. The error bars represent the standard deviation obtained with three replicates. Different capital letters (A, B, C, D, E, F) represent statistical differences between different storage cycles (*p* < 0.05).

The evolution of TVC and PTC are presented in Figure 1a,b.

For both TVC and PTC, a significant increase (*p* < 0.05) was observed during storage that could be explained by the metabolic activities of the microorganisms in Sterlet samples [26]. The microbiological acceptability for the human consumption of fresh fish fixed to 7 log_10_ CFU/g in plate count analysis. The initial TVC of the Sterlet sample was 2.27 log_10_ CFU/g, in agreement with the findings of Cheng et al. [3] who noted 3–4 log_10_ CFU/g as the TVC of Russian sturgeon fillets. The TVC increased significantly (*p* < 0.05) with the storage time, attaining 9.09 log_10_ CFU/g on day 18. From the obtained results, it could be concluded that the microbiological acceptability of Sterlet samples was fixed to 7 days, since the TVC was 7.10 log_10_ CFU/g.

The PTC of Sterlet on day 1 was 2.18 log_10_ CFU/g and reached 9.15 log_10_ CFU/g after 18 days of storage (Figure 1b). The maximum acceptable level of PTC was observed after 7 days of storage, in agreement with the results obtained with the TVCs in the present study and those of Bahram et al. [26], who observed the limit of acceptability of Beluga sturgeon on day 8.

### 3.2. Sensory Analysis

The overall acceptability of Sterlet samples during storage at 4 °C is shown in Figure 1c. The overall acceptability decreased significantly with the storage time, changing from 40 to 4.87 on day 1 and 18, respectively. Based on sensory analyses, the shelf-life of Sterlet samples was fixed to 7 days, in agreement with the results obtained with the TVCs and PTCs in the present study, and those of Cheng et al. [3], who noted a shelf-life of 7 days for Russian sturgeon (*Acipenser gueldenstaedti*) stored at 4 °C.

### 3.3. Physicochemical Measurements

The initial water content of the Sterlet sample on day 1 was 72.74 g/100 g, similar to the findings of Boughattas et al. [16] and Gharibzahedi & Mohammadnabi [27], who noted a water content of 73.90 and 75.75 g/100 g for Beluga and Sturgeon samples, respectively, (Figure 1d). A slight increase in the water content was noted during 18 days of storage, since a significant difference (*p* < 0.05) was observed between samples aged 1 day from those aged 7, 14, and 18 days. The obtained results are in agreement with the findings of Sanjuás-Rey, García-Soto, Barros-Velázquez, Fuertes-Gamundi, & Aubourg [28], who pointed out a slight increase in water content during storage of blue whiting (*Micromesistius poutassou*), since fresh samples (aged 1 day) presented a water content of 79.70 g/100 g, while those aged 9 days had a water content of 83.10 g/100 g. The oxidative rancidity of Sterlet samples is recognised as a major cause of the deterioration of the quality of fish during storage. Changes in the PV of the Sterlet are depicted in Figure 1e. The initial PV was 4.17 meq/kg of fish, which changed to 4.90 meq/kg of fish on day 4 and might have been ascribed to the formation of hydroperoxides in fish samples [29,30,31]. The maximum PV was attained on day 4 and then decreased due to the decomposition of the PV to secondary products [32].

The change in the TBARS values of the Sterlet sturgeon as a function of storage time is shown in Figure 1f. The initial value of TBARS was 0.03 mg MDA eq/kg of Sterlet, quite similar to the findings of Cheng et al. (2020) [3] and Bahram et al. (2016) [26], who depicted TBARS values of 0.01 and 0.02 mg MDA eq/kg for Russian sturgeon and Beluga sturgeon, respectively. An increase in TBARS value was observed during the first 11 days of storage (0.13 mg MDA eq/kg of Sterlet samples). This increase could be due to the decomposition of hydroperoxides into the secondary oxidation products, such as aldehydes [33]. Starting on the 11th day of storage, a decrease in the level of TBARS values was observed, with a value of 0.0926 mg MDA eq/kg of Sterlet attained on day 18. This decrease could be explained by the (i) loss of the secondary oxidation products formed, particularly the unstable low molecular weight volatile compounds; and/or (ii) the interaction between the TBARS and proteins [34]. On the other hand, Piranavatharsan, Jinadasa, and Jayasinghe, 2023 [35] observed a change in TBARS values in fresh Indian mackerel (Rastrelliger kanagurta). In their study, TBARS values were depicted at a level of 2.67 nmol/g on the first day of storage and increased to 11.80 nmol/g on the fifth day, attaining values of 14.33 nmol/g on the sixth day of storage. In another study, Papastergiadis et al. (2012) [36] used high-performance liquid chromatography (HPLC) to determine TBARS values in herring and rainbow trout samples kept at −27 °C. The authors found that TBARS values ranged from 0.30 ± 0.04 μg/g of herring on day 0 to 14.42 ± 1.46 μg/g of herring on day 159. A similar trend was observed for rainbow trout; the same method revealed that TBARS values increased from 0.09 ± 0.02 on day 0 to 1.53 ± 0.05 μg/g of rainbow trout on day 176. These results indicated that the reliability of the TABRS method varies according to the fish species.

### 3.4. Colour Measurements

The colour of fish plays a decisive role from a consumer point of view. Indeed, colour stability during storage is an important parameter and is related to the biochemical change in the fish muscle, pigment concentration, and spoilage level [19]. The changes in the colour of the Sterlet throughout storage are shown in Table 1. The lightness (L*) value of the Sterlet aged 1 day was 54.85, which decreased during the first 14 days of storage and then increased, attaining a value of 55.55 on day 18. The slight change in L* value might be due to greater water deposits at the Sterlet’s surface [37]. A similar trend was noted for whiteness (WI) values, in agreement with the findings of others [16].

The a* values displayed an increase during storage that grew from 7.15 to 10.73 for Sterlet samples aged 1 and 18 days, respectively. A general increase in a* value was observed during storage, except for in samples aged 7 days. The increase in a* values could be ascribed to the protein denaturation of the fish muscle [38].

The highest values of b* and *YI* were obtained for the samples aged 11 days. The yellow colour could be associated with lipid oxidation, as supported by the increase in TBARS values. To obtain more information about the colour parameters during storage, total colour difference (*ΔE*) was determined. The *ΔE* presented a general tendency to increase during the storage of Sterlet samples, which could be attributed to the oxidation process of lipids and proteins [39].

### 3.5. Global Analyses of the Physicochemical, Microbiological, and Colour Measurements

**Figure 2:**
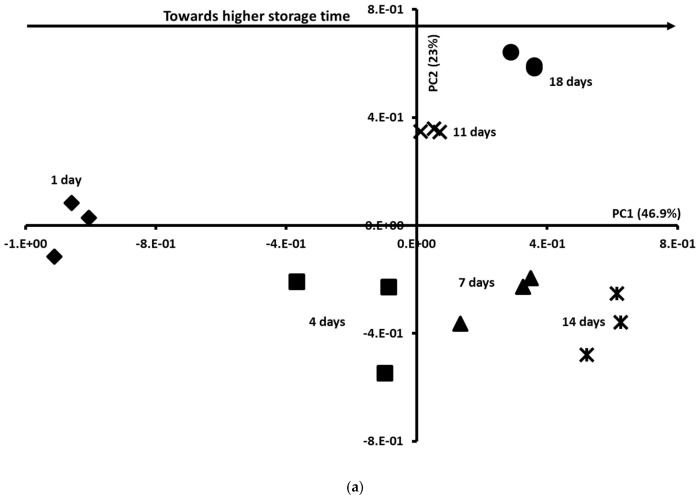

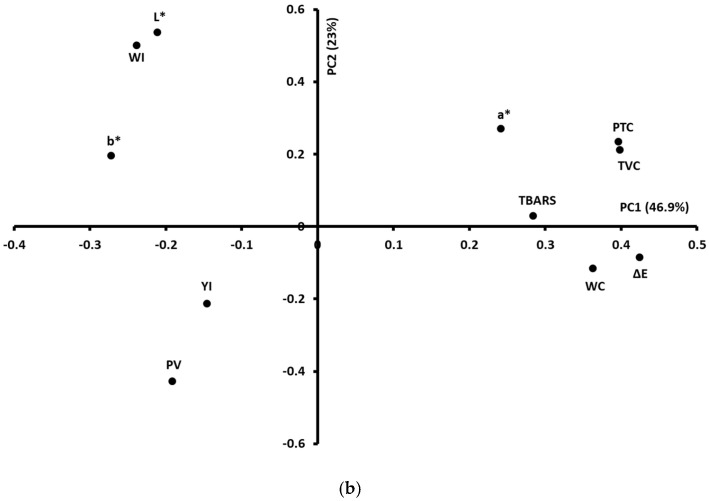
(**a**) Similarity map of the principal component analysis (PCA) determined by the principal components 1 (PC1) and 2 (PC2) of the physicochemical, colour and microbiological parameters assessed after 1 day (◆), 4 days (■), 7 days (▲), 11 days (✕), 14 days (✱) and 18 days (●) and (**b**) correlation chart of variables.

To extract information from the data sets, PCA was applied to the normalised physicochemical, microbiological, and colour data tables. Figure 2a showed the PCA similarity map defined by PCs 1 and 2 (46.9% and 23% of the total variance, respectively). A clear discrimination of the Sterlet samples according to storage time was observed, since according to the PC1, Sterlet samples aged 1 and 4 days exhibited negative score values, while those aged 7, 11, 14, and 18 days showed positive score values.

To investigate the discrimination between Sterlet samples during the storage time, the correlation circle was studied (Figure 2b). According to the PC1, Sterlet samples aged 14 and 18 days were characterised by a higher TVC, PTC, and TBARS value, while those aged 1 day presented the lowest values. The obtained results are in agreement with the observation of Hassoun & Karoui [29], who reported an increase in the TBARS values of whiting (*Merlangius merlangus*) fillets during 15 days of storage at 4 °C. The PC2 showed higher values of L* and WI for Sterlet samples aged 1 day.

### 3.6. Mid-Infrared Measurements

**Figure 3:**
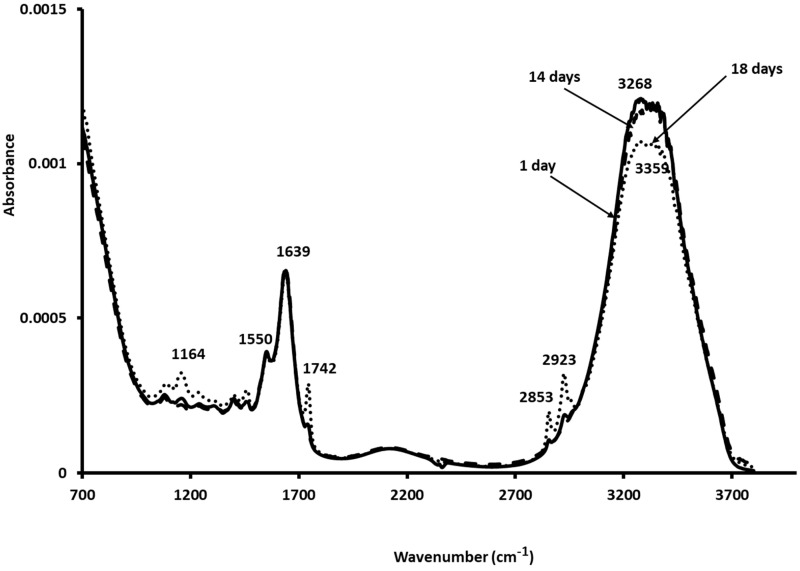
Normalised MIR spectra scanned on Sterlet samples stored at 4 °C for up to 18 days.

The 4000–700 cm^−1^ spectral region was determined on the Sterlet samples during 18 days of storage. Figure 3 shows the absorbance bands ∼1164, 1550, 1639, 1742, 2853, 2921, 3268, and 3359 cm^−1^. The absorbance at 1164 cm^−1^ could be ascribed to C-O and C-C stretching modes [39]. That observed at 1550 cm^−1^ (Amide II) characterised the N-H bending vibration. The absorbance at 1639 cm^−1^ characterised the C=O stretching vibration.

The protein was subjected to oxidation during the storage time that induced some changes in α-helix, β-sheet, β-turn, and random coil levels that had absorption at 1650–1660 cm^−1^, 1600–1640 cm^−1^, 1660–1690 cm ^−1^, and 1640–1650 cm^−1^, respectively.

Table 1b shows the evolution of the secondary structure of myofibrillar protein of the Sterlet samples during storage. The initial proportion of α-helix determined on samples aged 1 day was 11.83%, which decreased throughout storage, attaining 11.00% on day 18. A similar trend was observed for β-turn, since it changed from 47.06% for the fresh samples aged 1 day to 46.06% for those aged 18 days. An opposite trend was observed for the random coil level, since it varied from 7.36% to 8.10%. The obtained results are in agreement with Wang, He, Zhang, Chen, & Li [38], who observed the same trend in rabbit meat muscle during the freeze–thaw cycles. The Sterlet samples presented three strong bands at ~1742, 2853, and 2923 cm^−1^. The absorbance around 1742 cm^−1^ could be related to the formation of hydroperoxides during fish lipid oxidation; this trend was consistent with the physicochemical results for which low peroxide values were recorded on day 11. Furthermore, physicochemical analyses showed that oxidation parameters were influenced by storage time. The variation in MIR spectra during the storage period followed the same trend as the microbiological results; from these results, it appeared that MIR spectroscopy could be used as a rapid method for monitoring Sterlet freshness. The spectra located at ∼2923 and 2853 cm^−1^ were ascribed to methylene anti-symmetric and symmetric stretching modes, respectively. The band at ~3268 cm^−1^ was characteristic of proteins (N-H stretching) that decreased on day 18, while that observed at ~3359 cm^−1^ was due to the OH stretching of water [39].

### 3.7. Fluorescence Spectroscopy Measurements

**Figure 4:**
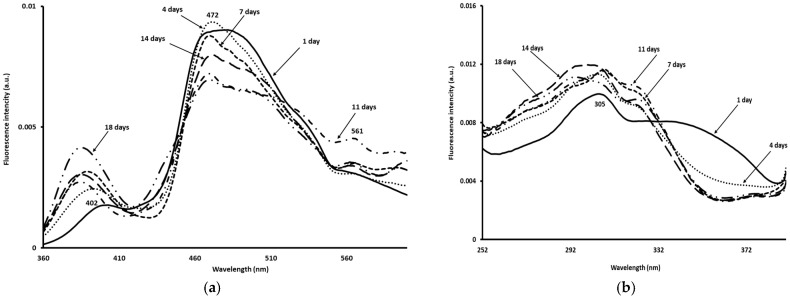

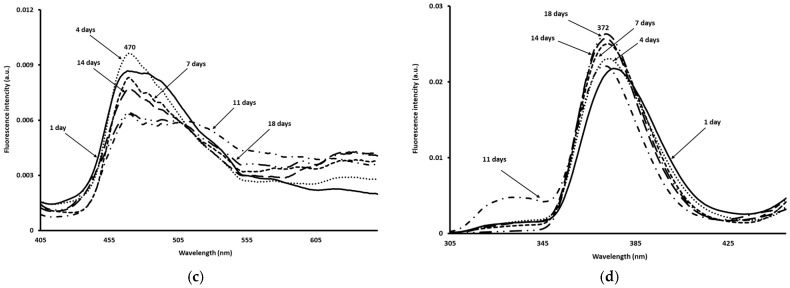
Normalised fluorescence emission spectra of (**a**) NADH (excitation: 340 nm, emission: 360–600 nm), (**b**) vitamin A (excitation: 250–390 nm, emission: 410 nm), (**c**) riboflavin (excitation: 380 nm, emission: 405–650 nm), and (**d**) tryptophan residues (excitation: 290 nm, emission: 305–450 nm) recorded on Sterlet samples stored at 4 °C for up to 18 days.

The normalised NADH spectra recorded after excitation set at 340 nm on the Sterlet samples during 18 days of storage are shown in Figure 4a.

These spectra exhibited three maxima located at ~385–402 nm, 472 nm, and 561 nm. The fresh Sterlet samples aged 1 day and 4 days presented the lowest fluorescence intensity at ~402 and 472 nm. This low fluorescence intensity could be attributed to a progressive oxidation of NADH to NAD+, a direct reflection of the loss of freshness and the metabolic alteration of the fish’s muscle tissue. These biochemical reactions (NADH oxidation, among others) were accompanied by other metabolic degradations, including a drop in ATP, the formation of compounds responsible for undesirable tastes and odours (such as hypoxanthine), and increased lipid oxidation [23,40]. These results were consistent with the physicochemical measurements, as PVs were higher on day 1 and 4, indicating primary oxidation products. On the contrary, samples aged 18 days showed the highest fluorescence intensity at ~402 nm and the lowest one at ~472 nm. This result was in agreement with the findings of Boughattas et al. [16], who reported similar findings after excitation set at 340 nm for Russian sturgeon. These changes might be explained by the oxidation of the cell cytoplasm during storage, leading to the transformation of NADH to NAD^+^ and a change in the concentration, thus modifying the shape of the fluorescence spectra. It appeared that the shape of the NADH emission spectra was correlated with the freshness state of Sterlet samples. Indeed, a high correlation was obtained between TVC, PTC, and the fluorescence intensity at 402 nm (R^2^ = 0.77 and 0.81, respectively). The fluorescence intensities of the NADH spectra at 402 (R^2^ = 0.69) and 472 nm (R^2^ = 0.70) were found to be correlated with the overall acceptability of the Sterlet samples. From the obtained results it could be concluded that the NADH fluorescence spectra could be used as a fingerprint for freshness identification, in agreement with other investigations reporting that NADH spectra could be used for the freshness identification of Atlantic mackerel fillets [18], whiting fish samples [17], and sea bass fillets [19], among others.

Figure 4b displays the normalised fluorescence spectra acquired after emission set at 410 nm for the Sterlet samples. These spectra exhibited a maximum of ~305 nm. The excitation spectra of the vitamin A scanned on the Sterlet showed some differences as a function of the storage time. Indeed, fresh samples aged 1 day showed the lowest fluorescence intensity at 305 nm, while those aged 7, 11, and 14 days presented the highest fluorescence intensity at 305 nm. This could be due to the crystallisation of fat throughout storage inducing an increase in the fluorescence intensity at 305 nm.

Figure 4c depicts an example of the normalised riboflavin fluorescence spectra recorded on Sterlet samples during 18 days of storage at 4 °C. Indeed, a decrease in the fluorescence intensity ~470 nm was observed with the increase in the storage time. This decrease could be attributed to the fact that riboflavin can degrade under the influence of light, oxygen, and temperature, forming products such as lumichrome, which has a different fluorescence. This degradation results in a decrease in the fluorescence intensity of riboflavin and/or a change in the emission spectrum, which could indicate a degradation of the quality of Sterlet samples, as it reflects the oxidative and photochemical alteration of the vitamin compounds. The peak at ~470 nm could be ascribed to the lipid oxidation products and compounds formed following the reaction of aldehydes with proteins or amino acids [40]. The riboflavin was degraded into a fluorescent product, namely lumichrome, with the emission maximum located between 444 and 479. Thereby, the peak at ~470 nm could be ascribed to the photo breakdown product from riboflavin.

The normalised tryptophan spectra recorded after excitation set at 290 nm on Sterlet samples kept at 4 °C over 18 days are displayed in Figure 4d. Fresh samples exhibited a maximum at ~372 nm which could be ascribed to the maximum emission of tryptophan. An increase in the fluorescence intensity at ~372 nm with the storage time was noted, which could be due to protein–protein interactions and different myofibril structures, in agreement with the other findings [17,31].

### 3.8. Storage Oversight Using Mid-Infrared and Fluorescence Data by Applying Chemometric Tools: PCA and FDA

**Figure 5:**
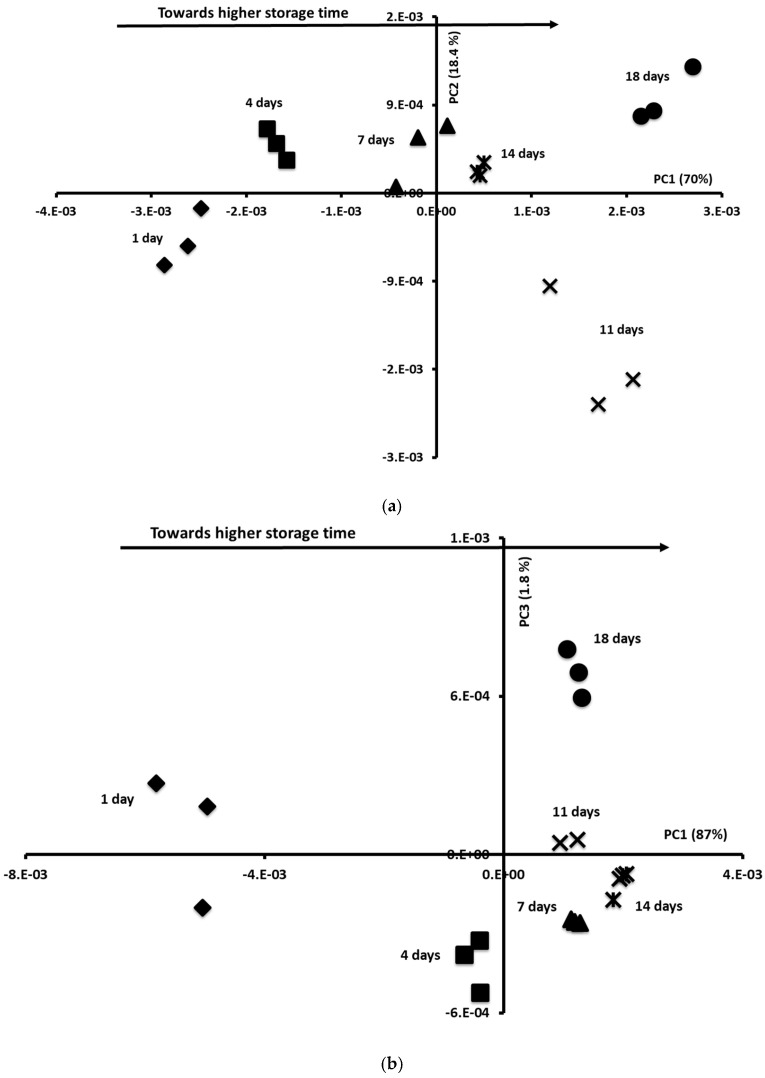

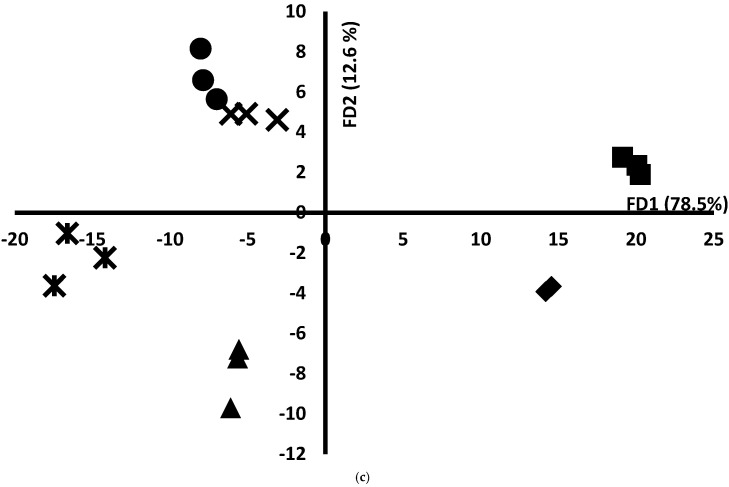
Similarity map of the principal component analysis (PCA) determined by the principal components (**a**) 1 (PC1) and 2 (PC2) performed on the NADH fluorescence spectra and (**b**) 1 (PC1) and 3 (PC3) performed on the vitamin A fluorescence spectra. (**c**) Similarity map of the factorial discriminant analysis (FDA) performed on the first five PCs of the PCA applied to mid-infrared spectra recorded on Sterlet samples aged 1 day (◆), 4 days (■), 7 days (▲), 11 days (✕), 14 days (✱), and 18 days (●).

To extract information from the spectral data sets, PCA was applied, separately, to each fluorescence spectra and MIR spectra. The NADH and vitamin A spectra showed the most efficient discrimination between Sterlet samples as a function of their storage time. Indeed, NADH and vitamin A spectra are found to be more effective than riboflavin in discriminating fish samples during storage, due to their chemical properties and specific deterioration dynamics; vitamin A is directly involved in lipid oxidation reactions, a key marker of fish degradation, as well as in the protein–lipid and lipid–lipid interactions. Its degradation generates volatile compounds (such as aldehydes) that are easily detected by fluorescence spectroscopy. NADH reflects the residual metabolic activity of tissues: its fluorescence intensity decrease correlates with loss of freshness and microbial proliferation [23]. Regarding riboflavin/tryptophan spectra, it should be noted that riboflavin, being less sensitive to storage conditions, offers a less variable spectral signal. This difference could be attributed to the nature of the dominant biochemical reactions during storage (lipid oxidation and enzymatic activity) and the ability of the markers to reflect these changes measurably in fish samples. Indeed, the similarity map of the PCA performed on the NADH fluorescence spectra defined by PCs 1 and 2 (70% and 18.4% of the total variance, respectively) allowed for the clear differentiation of the samples according to their storage time (Figure 5a).

According to the PC1, Sterlet samples aged 1, 4, and 7 days presented mostly negative score values, whereas the other samples exhibited positive scores. These results were in agreement with those obtained with microbiological and sensory analyses which indicated that the shelf-life of the Sterlet samples was fixed to 7 days. It could be concluded that the NADH spectra could be used for the determination of the shelf-life of Sterlet samples. The similarity map of the PCA applied to the vitamin A fluorescence spectra allowed for the discrimination of samples aged 1 and 4 days with negative scores according to PC1 (87% of the total variance) from the others (Figure 5b). Similar results were obtained with MIR spectra.

In a second step, FDA with leave-one cross-validation was applied to the first five PCs of the PCA applied to the MIR and fluorescence spectra. The map of the leave-one cross-validation data set defined by the first two-discriminant factors of the FDA applied to MIR is shown in Figure 5c. Samples aged 1 and 4 days presented positive scores according to FD1, accounting for 78.5%, while the other samples exhibited negative scores. These results suggest that the MIR data sets may be a potential approach for recognising Sterlet samples according to their storage time. Indeed, correct classification with leave-one cross-validation was observed for 94.44% (Table 1c). This table shows that samples aged 1, 4, 7, 11, and 18 day(s) were 100% correctly classified. Only 1 sample, aged 14 days, was classified as belonging to the group aged 7 days. Similar results were obtained with NADH samples, since a correct classification of 94.44 was noted.

### 3.9. Correlation Between Structure and Physicochemical and Microbiological Properties of PPI

**Figure 6:**
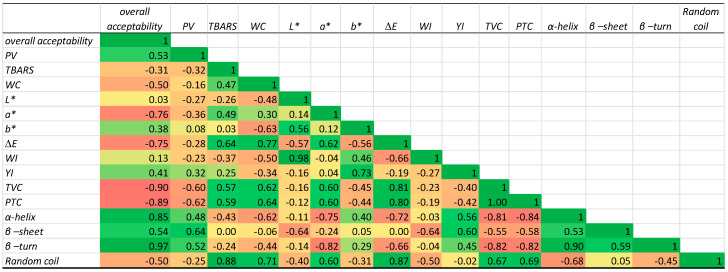
Heatmap of the Pearson correlation coefficients of Sterlet samples’ sensory, physicochemical, microbiology, and structural properties. The colour scale ranges from dark red (strong negative correlation, −1) to dark green (strong positive correlation, 1). Parameters include structural properties (β-sheet, α-helix, β-turn, random coil, overall acceptability, peroxide value (PV), total viable counts (TVCs), psychrotrophic counts (PTCs), water content (WC), thiobarbituric acid reactive substances (TBARS), L*, a*, b*, *ΔE*, *WI*, and *YI*.

A correlation analysis was performed between the structural, physicochemical, sensory and microbiology parameters (Figure 6).

Overall acceptability showed a strong positive correlation with α-helix (0.85) and β-turn (0.97) and a negative correlation with microbiological parameters (TVC: −0.90 and PTC: −0.89) and colour parameters (a*: −0.76 and *ΔE*: −0.75). These observations highlight the involvement of different factors in determining the overall acceptability of Sterlet samples, especially the development of TVCs and PTCs. Similarly, the PV showed strong negative correlations with the microbiology parameters TVC (−0.60) and PTC (−0.62), while TBARS exhibited a strong positive correlation with random coil (0.88). This could be explained by the fact that the development of microorganisms delayed the oxidation of Sterlet samples. A strong positive correlation was obtained between TVC and PTC (1) indicating that the development of the TVC induced an increase in the PTC. Strong negative correlations were also observed between the TVC and α-helix (−0.81) and β-turn (−0.82); similar results were obtained with the PTC, since high correlations with α-helix (−0.84) and β-turn (−0.82) were noted. It could be concluded that the development of the PTC and TVC disorganised the structure of α-helix and β-turn structures. It should be kept in mind that the observed correlations do not imply direct causality between the structural observations at the molecular level and microbial parameters.

## 4. Conclusions

Different analytical techniques were used to monitor changes occurring in Sterlet samples throughout storage for up to 18 days at 4 °C. Overall results indicated an increase in the water content, TBARS, TVCs, and PTCs during storage. These changes at the macroscopic level induced some modifications at the molecular level, since a decrease in the levels of α-helix and β-turn were observed during storage. The sensory and microbiological analyses showed that Sterlet samples were considered unacceptable for human consumption after 7 days of storage. These results were confirmed by the PCA applied on the NADH spectra, since samples aged 7 days or less were well separated from the others. The development of PTC and TVC disorganised the structure of the α-helix and β-turn structures determined by mid-infrared spectroscopy.

NADH fluorescence spectra as well as MIR spectra were found to be considered promising, reliable, and non-invasive tools for monitoring Sterlet freshness, which could be used as rapid tools for screening fish freshness. These techniques should be integrated into a battery of complementary tests for a comprehensive assessment of fish freshness during storage.

## Figures and Tables

**Table 1:** Change in colour parameters (**a**), the secondary structure determined by mid-infrared (**b**), and (**c**) the classification table of the FDA with leave-one cross-validation of mid-infrared and NADH spectra of Sterlet samples stored at 4 °C for up to 18 days.

(**a**)								
**Parameters**	**Storage Time (day)**							
	**1**	**4**		**7**	**11**		**14**	**18**
L*	54.85 ± 0.10 ^B^	52.62 ± 0.51 ^C^		51.78 ± 0.12 ^D^	54.42 ± 0.07 ^B^		50.68 ± 0.04 ^E^	55.55 ± 0.16 ^A^
a*	7.15 ± 0.15 ^D^	9.54 ± 0.09 ^C^		6.91 ± 0.10 ^D^	10.16 ± 0.06 ^B^		10.20 ± 0.07 ^B^	10.73 ± 0.12 ^A^
b*	14.80 ± 0.03 ^A,B^	14.58 ± 0.03 ^B^		13.39 ± 0.37 ^D^	15.22 ± 0.05 ^A^		13.64 ± 0.05 ^C,D^	13.93 ± 0.04 ^C^
***ΔE***	0.00 ± 0.00 ^D^	3.20 ± 0.19 ^C^		3.40 ± 0.19 ^C^	3.08 ± 0.13 ^C^		5.30 ± 0.12 ^A^	3.81 ± 0.08 ^B^
***WI***	51.94 ± 0.13 ^A^	49.53 ± 0.46 ^C^		49.47 ± 0.04 ^C^	50.87 ± 0.04 ^B^		47.81 ± 0.06 ^D^	52.17 ± 0.19 ^A^
***YI***	38.56 ± 0.15 ^B^	39.58 ± 0.32 ^A,B^		36.94 ± 0.93 ^C^	39.96 ± 0.7 ^A^		38.45 ± 0.17 ^B^	35.83 ± 0.21 ^C^
(**b**)								
**Secondary Structure**	**Storage Time (day)**							
	**1**	**4**		**7**	**11**		**14**	**18**
**α-helix (%)**	11.83 ± 0.46 ^A^	11.5 ± 0.10 ^A,B^		11.5 ± 0.10 ^A,B^	11.46 ± 0.05 ^A,B^		11.36 ± 0.05 ^A,B^	11.00 ± 0.00 ^B^
**β-sheet (%)**	33.76 ± 0.63 ^A^	34.33 ± 0.45 ^A^		33.66 ± 0.68 ^A^	33.46 ± 0.23 ^A^		33.96 ± 0.28 ^A^	33.08 ± 0.40 ^A^
**β-turn (%)**	47.06 ± 1.96 ^A^	46.96 ± 1.51 ^A^		46.93 ± 0.68 ^A^	46.60 ± 0.95 ^A^		46.33 ± 0.63 ^A^	46.06 ± 1.10 ^A^
**Random coil (%)**	7.36 ± 1.67 ^A^	8.63 ± 0.61 ^A^		7.96 ± 0.90 ^A^	8.13 ± 0.37 ^A^		8.20 ± 0.43 ^A^	8.10 ± 0.60 ^A^
(**c**)								
**Predicted/Observed**		**Storage Time (day)**						
		**1**	**4**	**7**	**11**	**14**	**18**	**% of Correct Classification**
		**Mid-infrared spectra**						
**1 day**		3	0	0	0	0	0	100.00%
**4 days**		0	3	0	0	0	0	100.00%
**7 days**		0	0	3	0	0	0	100.00%
**11 days**		0	0	0	3	0	0	100.00%
**14 days**		0	0	1	0	2	0	66.67%
**18 days**		0	0	0	0	0	3	100.00%
**Total**		3	3	4	3	2	3	94.44%
		**NADH fluorescence emission spectra**						
**1 day**		3	0	0	0	0	0	100.00%
**4 days**		0	3	0	0	0	0	100.00%
**7 days**		0	0	3	0	0	0	100.00%
**11 days**		0	0	0	2	0	1	66.67%
**14 days**		0	0	0	0	3	0	100.00%
**18 days**		0	0	0	0	0	3	100.00%
**Total**		3	3	3	2	3	4	94.44%

Mean values and standard deviations from three replicates are presented. Different capital letters (A, B, C, D, E)) represent statistical differences between different storage days (*p* < 0.05). Mean values and standard deviations from three replicates are presented. Different capital letters (A, B) represent statistical differences between different storage days (*p* < 0.05).

## Data Availability

The original contributions presented in the study are included in the article, further inquiries can be directed to the corresponding author.
